# History and origin of the HIV-1 subtype C epidemic in South Africa and the greater southern African region

**DOI:** 10.1038/srep16897

**Published:** 2015-11-17

**Authors:** Eduan Wilkinson, Susan Engelbrecht, Tulio de Oliveira

**Affiliations:** 1 Division of Medical Virology, Faculty of Medicine and Health Sciences, Stellenbosch University, Tygerberg, Cape Town, Western Cape Province, South Africa; 2 Africa Centre for Health and Population Studies, University of KwaZulu-Natal, Mtubatuba, KwaZulu-Natal, South Africa; 3 National Health Laboratory Services, Tygerberg Academic Hospital, Tygerberg Coastal, Cape Town, South Africa; 4 School of Laboratory Medicine and Medical Sciences, Nelson R Mandela School of Medicine, College of Health Sciences, University of KwaZulu-Natal, Durban, South Africa

## Abstract

HIV has spread at an alarming rate in South Africa, making it the country with the highest number of HIV infections. Several studies have investigated the histories of HIV-1 subtype C epidemics but none have done so in the context of social and political transformation in southern Africa. There is a need to understand how these processes affects epidemics, as socio-political transformation is a common and on-going process in Africa. Here, we genotyped strains from the start of the epidemic and applied phylodynamic techniques to determine the history of the southern Africa and South African epidemic from longitudinal sampled data. The southern African epidemic’s estimated dates of origin was placed around 1960 (95% HPD 1956–64), while dynamic reconstruction revealed strong growth during the 1970s and 80s. The South African epidemic has a similar origin, caused by multiple introductions from neighbouring countries, and grew exponentially during the 1980s and 90s, coinciding with socio-political changes in South Africa. These findings provide an indication as to when the epidemic started and how it has grown, while the inclusion of sequence data from the start of the epidemic provided better estimates. The epidemic have stabilized in recent years with the expansion of antiretroviral therapy.

HIV/AIDS is one of the most serious health problems facing humanity today. Sub-Saharan Africa bears the brunt of the global HIV-1 burden with the southern African region accounting for roughly one third of all infections globally^1^. The spread of the epidemic in southern Africa happened at an extremely accelerated rate throughout the 1980s, 90s and 2000s while HIV prevalence rates in other areas of the African continent have grown far more slowly^1^.

Until fairly recently, standard epidemiological methods were the only way to investigate the history of HIV epidemics. However, in the past decade, advances in coalescence and molecular clock methods have allowed the inference of the origin and growth of HIV epidemics from genetic data. To date, phylogenetic and phylodynamic methods have successfully been employed in the study of HIV-1 subtype C epidemics in a range of different countries, such as Malawi^2^, Brazil^3^, Zimbabwe^4^, Ethiopia^5^, the United Kingdom^6^, India^7^, Senegal^8^ and Angola^9^. A number of other studies have investigated the history of the global HIV-1 subtype C pandemic^2^,^10^,^11^, and two others have investigated the regional HIV-1 subtype C epidemic in East Africa^12^ and South America^13^ respectively.

These studies have provided valuable insight into the origin and epidemic spread of the HIV-1 subtype C strain in different geographical areas. However, most of them used only contemporary strains and to date no in-depth phylogenetic investigation has been conducted on sequence data sampled from the start of the epidemic in South Africa. Furthermore, a comprehensive reconstruction of the entire southern African region and of the South African HIV-1 subtype C epidemic was still lacking. The need for such a study is ever more pressing considering the high prevalence within the region and within South Africa.

In this study, we took an in-depth look at the evolutionary history of the heterosexual HIV-1 subtype C epidemic in South Africa through the analysis of longitudinally sampled sequence data with the application of coalescence and molecular clock methods. This study was conducted to ascertain: (i) when the epidemic originated in South Africa and southern Africa and (ii) what the dynamic growth aspects of the epidemic were. In addition, we briefly discuss the results within the unique historical context of the area (i.e. Apartheid, independence movements, civil wars, labour related migration and other cultural factors) and highlight their potential influence on the origin and growth of the epidemic in the country and in the region.

## Materials and Methods

### Ethical considerations

This study (Reference number N09/08/221) was approved by the Health Research Ethics Committee (HREC) of Stellenbosch University (IRB0005239). The old samples dating from 1989 to 1992 were obtained with a waiver of informed consent. The HREC complies with the South Africa National Health Act No 612003 and the United States code of Federal Regulations title 45 Part 46. The committee also abides by the ethical norms and principles for research established by the Declaration of Helsinki and the Guidelines of the South African Medical Research Council and the South African Department of Health. All of the methods described in this manuscript were carried out in accordance with the approved guidelines of HREC.

### Genotyping of archived Cape Town specimens

The first viral isolation of HIV-1 in South Africa was performed within the Division of Medical Virology, at Stellenbosch University, Tygerberg, Cape Town, South Africa^14^, which became a repository of samples for HIV testing from across the country in the early years of the HIV epidemic. To date, the Division has a sample repository containing more than 40,000 samples. In order to identify potential subtype C infected individuals from the start of the epidemic in South Africa, we evaluated all diagnostic and research archives at the Division. Two cohorts from the early years of the epidemic were identified as containing subtype C isolates. The first cohort contained 218 HIV positive isolates that were sampled between 1985 and 1990. The 218 isolates were previously serotyped with the V3 peptide enzyme immunoassay (cPEIA) from Roche^15^. In total, 27 isolates were identified as subtype C, 62 as subtype B, 1 isolate as subtype D, 3 isolates as subtype A, 14 as complex or potential recombinants, while the remaining 111 isolates tested inconclusively for a single subtype with the cPEIA serological assay. The second cohort contained 93 isolates that were sampled between 1989 and 1992. These 93 isolates were previously genotyped through envelope-V3 sequencing [personal communication S Engelbrecht]. In total, 87 of the samples were genotyped based on their V3 sequences as subtype C, 5 as subtype B and 1 as subtype A1. We selected the 27 isolates that tested positive for subtype C with the cPEIA assay and the 87 isolates that were genotyped as subtype C for further *gag* p24 and *pol* genotyping. Of the 114 isolates that were targeted for genotyping, only 25-*gag* p24 and 45-*pol* genotypes could be generated. The low success rate for genotyping can be attributed to the age of these samples and a degree of nucleic acid degradation following almost 20 years of storage.

Briefly, nucleic acid was extracted with the use of the QIAamp DNA Blood Mini Kit (QIAGEN, Dusseldorf, Germany) according to the manufacturers proposed protocols. All DNA samples were stored at 4 °C until further use. Two loci of the HIV-1 genome were targeted for genotyping: the *gag* p24 (1246–1727 bp relative to HXB2) and a partial *pol* fragment, including the entire *protease* and a large segment of the *reverse transcriptase* coding region of the *polymerase* gene (2246–3321 bp relative to HXB2). The methodology and primers that were used for the amplification and sequencing of target genes from DNA or cDNA templates, were adopted from Swanson and co-workers^16^ for the *gag* p24 assay and from Plantier and co-workers^17^ for the *pol* assays. The sequencing primers for the *pol* assay were complemented with the use of Pol 1D (5′-TCC CTC AAA TCA CTC TTT GGC-3′) and *pol* 3 (5′-GGG GGA TGC ATA TTT TTC AG-3′). The *gag* and *pol* regions were chosen due to the fact that they had successfully been used in the past to infer evolutionary histories^2^,^3^,^4^,^5^,^6^,^7^,^8^,^9^,^10^.

Newly sequenced isolates were checked for sequence quality and possible contamination with the use of the HIV-1 sequence quality control tool (http://bioafrica.mrc.ac.za/tools/pppweb.html) from the BioAfrica website.

### Sampling strategy: complementing data sets with published sequences

To infer the origins of the epidemics in South Africa and countries in southern Africa, we retrieved all full-length subtype C sequences (n = 375) that had been sampled in the region from Los Alamos HIV-1 sequence database (http://www.hiv.lanl.gov/content/index). For the purpose of this study, the southern African region includes the following countries: Angola, Botswana, Lesotho, Malawi, Mozambique, Namibia, South Africa, Swaziland, Zambia and Zimbabwe. The full-length HIV-1 subtype C sequences were concatenated into two segments covering the *gag* p24 (1258–1701 relative to HXB2) and *pol* (2244–3245 relative to HXB2) coding regions. In addition, all homologous *gag* p24 (1258–1701 relative to HXB2) and *pol* (2244–3245 relative to HXB2) sequences that were sampled prior to the year 2000 were retrieved from the LANL HIV-1 sequence database in order to provide a better understanding of genetic diversity through time and to reduce uncertainty^18^.

### HIV subtyping

All sequences, both newly generated as well as those obtained through data mining, were submitted to two online HIV-1 subtyping tools, REGA v 3.0 (http://www.bioafrica.net/rega-genotype/html/subtypinghiv.html)^19^ and the jumping profile Hidden Markov Model or jpHMM (http://jphmm.gobics.de/submission_hiv)^20^, to screen for potential recombinants and to ensure that only HIV-1 subtype C isolates were included in any further analysis.

### Data set alignment

We inferred epidemic histories from longitudinal sequence data. Briefly, *gag* p24 (1258–1701 relative to HXB2) and *pol* (2244–3245 relative to HXB2) segments from the full-length data set were used to infer the epidemic in the southern African region. Additionally, we also created another two data sets with sequence data from specimens sampled prior to the year 2000 in order to provide a better understanding of diversity through time and to determine if the use of older sequences reduces uncertainty in parameter estimates.

These four data sets were aligned in ClustalW v 2.1 (http://www.clustal.org/download/current)^21^. Alignment files were then manually edited in Se-Al v 2.0 (http://tree.bio.ed.ac.uk/software/seal) until a perfect codon alignment was obtained. Due to the problem of HIV drug resistance and its adverse effects on phylogenetic inference, all sites associated with drug resistance mutation were excluded from the alignments.

### Molecular clock signal analysis

Maximum-Likelihood (ML) tree topologies were constructed for each of the alignments with the use of the HKY model of nucleic acid substitution^22^, an estimated Gamma shape parameter and the approximate likelihood ratio test method of branch support^23^ in PhyML v 3.0^24^. Trees were analysed in Path-O-Gen v 1.3 (http://tree.bio.ed.ac.uk/software/pathogen) to evaluate the temporal signal and general clocklikeness of each of the data sets, as well as to identify potential outliners that deviated from the standard regression. The molecular clock analysis of the two *gag* p24 data sets (i.e. whole genomes and whole genomes plus older sequences) placed the estimated time to the most recent common ancestor (tMRCA), based on the crude root-to-tip regression, at 1949.231 and 1960.881, respectively. The respective R squared values were 0.181 for the *gag* whole genome and 0.211 for the whole genome data set supplemented with old sequence data. Additionally, the substitution rates were estimated at 2.956 × 10^−3^ and 2.836 × 10^−3^ respectively. The estimated tMRCA for the *pol* data sets were placed at 1947.269 and 1951.869 for the full-length and complimented full-length *pol* data sets respectively, while the substitution rates were estimated at 2.331 × 10^−3^ and 2.051 × 10^−3^ respectively. Furthermore, the R^2^ values for the two data sets were 0.219 and 0.204 respectively. From the analyses of the various data sets in Path-O-gen, only one outlier (KF780974 Genbank accession number) was identified and removed in the subsequent analyses. The estimated dates and substitution rates were in range with previous evolutionary studies of HIV-1 subtype C, suggesting that there was clock like signal in the data for further molecular clock analysis.

### tMRCA estimation and epidemic reconstruction

Bayesian Markov Chain Monte Carlo (MCMC) analyses of the four different data sets were set up in BEAUti v 1.7.5, which is part of the BEAST software package^25^. The estimated tMRCAs of the various data sets were inferred with the use of two parametric (Constant Population Size and Exponential Growth) tree priors^26^,^27^ and three non-parametric tree priors (Bayesian Skyline Plot^28^, Bayesian Sky Grid^29^ and Sky Ride models^30^). Evolutionary histories were inferred under both relaxed and strict molecular clock assumptions. Substitution rates were estimated for selected runs while for other runs the substitution rates were fixed at 3.00 × 10^−3^ mutations/site/year for the *gag* p24 data sets and 2.55 × 10^−3^ mutations/site/year for the partial *pol* data sets based on the findings from previously published data^10^,^31^. In the manuscript, we present the results of the estimated rates, which were similar to the fixed rates used in the analyses.

Each of the epidemic reconstructions was performed with the implementation of the SRD06 model of nucleotide substitution^32^. For each demographic model, two independent runs of length 100 million steps were performed. Each run was executed in BEAST v 1.7.5 software application on a Linux supported computer cluster. Samples of trees and parameter estimates were collected every 10 000 iterations to build a posterior distribution of parameters. Convergence in each of the MCMC runs was assessed in Tracer v 1.5 (http://tree.bio.ed.ac.uk/software/tracer) on the basis of the effective sample size (ESS) and convergence in the trace files. Bayes factors (BF)^33^ were used for the selection of the best performing model following 1000 bootstrap replicates in Tracer v 1.5. The log and corresponding tree file were used for the reconstruction of the epidemic through Bayesian Skyline, SkyGrid and SkyRide reconstruction, as well as the estimation of the percentage lineages through time.

### Phylogenetic investigation to establish the evolutionary relationship of southern African subtype C strains

A phylogenetic investigation was conducted in order to investigate the evolutionary relationship of southern African HIV-1 subtype C isolates to other HIV-1 subtype C strains from around the world.

Homologous HIV-1 subtype C sequences were retrieved from the LANL HIV-1 sequence database (http://www.hiv.lanl.gov/) for the two different loci of the HIV genome: a *gag* p24 fragment (1246–1727 bp relative to HXB2) and a partial *polymerase* fragment (2246–3321 bp relative to HXB2). In total, 2242 *gag* p24 sequences and 5143 *pol* sequences were identified. Due to the over representation of *pol* sequences in the database we randomly selected ~50% of taxa from the 5143 *pol* sequences. Homologous sections of the HXB2 reference strain were included for the purpose of rooting phylogenies.

Following the exclusion of duplicate sequences from the same patient and the combination of the newly sequenced isolates from Cape Town, the two sequence data sets were aligned in Clustal W v 2.1 and edited as described previously. Two Maximum Likelihood (ML) tree topologies were inferred in phyML v 3.0^24^ and RAxML^34^. Maximum-likelihood tree topologies constructed in phyML were inferred with the SPR method of tree search optimization and the approximate likelihood ratio test (aLRT) method of branch support^23^, while ML-tree branch support constructed in RAxML was inferred with 100 bootstrap replicates^35^,^36^. Each of the newly constructed phylogenies was imported into FigTree v 1.3.1 (http://tree.bio.ed.ac.uk/software/figtree) and manually investigated. The clustering of southern African isolates with other sequences was assessed with the use of the PhyloType software application^37^. Clusters of five or more sequences from each country were identified with aLRT >0.9 and bootstrap >70%.

### Sequence information

All of the sequences that were generated from old specimens were submitted to GenBank with the following accession numbers: *gag* p24 (KF781071–KF781095) and *pol* (KF780965–KF781009). All alignment files and tree files can be made available on request from the authors.

## Results

### Data set construction

The genotyping of the 114 specimens, which were sampled prior to the year 1993, produced 25-*gag* p24 and 45-*pol* sequences. Sequence quality control measures that were performed identified no traces of sequence contamination amongst these new sequences. Furthermore, subtyping of these new strains with two online HIV subtyping methods identified all isolates as subtype C strains. No recombinants were detected in the analyses. Database searches for old homologous subtype C strains identified 25-*gag* p24 isolates and 5-*pol* isolates from the southern African region sampled in the previous century (<2000). The data mining of full-length HIV-1 subtype C isolates of southern African countries, which was retrieved from the Los Alamos National Laboratory (LANL) HIV-1 sequence database, produced 375 isolates. However, full-length subtype C sequences were only available for four southern African countries: South Africa, Zambia, Botswana and Malawi. The combination of these old isolates with sequence homologous sections from the full-length data set produced four sequence data sets, which were used for the estimation of epidemic histories. A full breakdown of these four data sets, as well as the years and countries of sampling are provided in Table 1.

### Molecular clock analysis of the origin of the southern African epidemic

The estimated tMRCA dates and nucleotide substitution rates for the southern African epidemic were inferred from four different data sets. Estimates were calculated in duplicate under various different evolutionary models of population growth using both strict and relaxed molecular clock assumptions. Parameter estimates were highly consistent across the different evolutionary models and duplicate runs. Based on BF scores, the models inferred under a relaxed molecular clock outperformed those inferred under a strict molecular clock assumption (BF = 12.21), while estimates inferred under an estimated substitution rate slightly outperformed those inferred under a fixed substitution rate (BF = 10.38). High effective sample sizes (>200) were recorded for all runs following 100 million steps in the Markov Chain, which demonstrates good mixing in the sampling chain.

**Figure 1:**
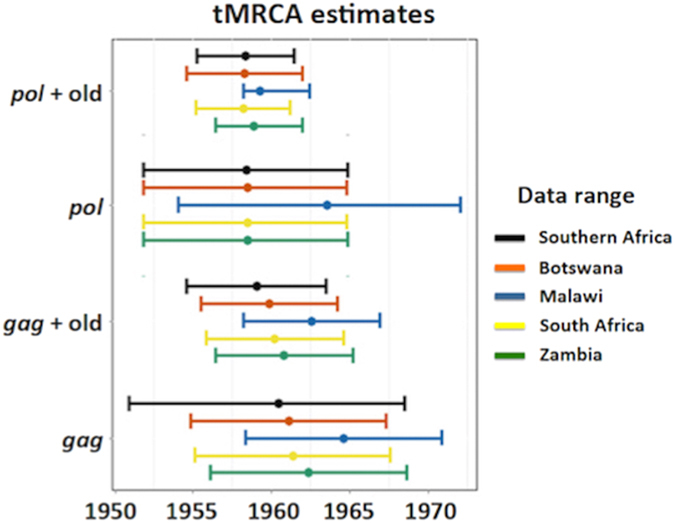
The estimated time to the most recent common ancestor for the southern African region as well as for several southern African countries, which were co-estimated in BEAST from the four different data sets. The dot represents the mean estimated date and the bars represent the 95% confidence interval (95% HPD).

The analyses of the two *gag* p24 data sets place the tMRCA for the entire southern African epidemic at 1960.432 (95% HPD 1950.874–1968.478) and 1959.035 (95% HPD 1954.598–1963.473) for the full-length and full-length + old sequence data sets, respectively (Table 2). Sub-regional tMRCA estimates, co-estimated from the full southern African *gag* p24 data sets, closely mirrored those of the full data sets, with a slight deviation in the estimates for the Malawian epidemic (Fig. 1). This can be attributed to the small sample size of Malawian sequences within the data sets (n = 14), which resulted in a slightly younger tMRCA for this sub-region. The mean estimated nucleotide substitution rates for the *gag* p24 data sets were 2.60 × 10^−3^ mutation/site/year (95% HPD 2.23 × 10^−3^ − 3.05 × 10^−3^) for the *gag* full-length data set and 2.64 × 10^−3^ mutation/site/year (95% HPD 2.41 × 10^−3^ − 2.90 × 10^−3^) for the *gag* full-length + old sequence data set. These rates are in close agreement with the estimated substitution rates found by Novitsky *et al.*, in the reconstruction of the global HIV subtype C pandemic, which were reconstructed from longitudinally sampled *gag* p24 sequences^10^.

Epidemic reconstruction from the *pol* data sets (full-length and full-length + old sequences) also showed a close congruency between methods and data sets (Table 2). The estimated tMRCA of the epidemic inferred from the original *pol* data set (only containing sequence information from the full-length isolates) placed the origin of the southern African epidemic around 1958.434 (95% HPD 1951.827–1964.901). Similarly, the estimate of the tMRCA inferred from the second *pol* data set, which included sequence information from older strains, placed the origin of the epidemic in the region around 1958.342 (95% HPD 1955.253–1961.431). Large congruency in the sub-regional tMRCA estimates was also observed in the *pol* data sets, with the exception of the Malawian estimate (Fig. 1). The mean estimated nucleotide substitution rates for the *pol* data sets were 2.21 × 10^−3^ mutation/site/year (95% HPD 1.99 × 10^−3^ − 2.46 × 10^−3^) for the *pol* full-length data set and 2.15 × 10^−3^ mutation/site/year (95% HPD 1.95 × 10^−3^ − 2.44 × 10^−3^) for the *pol* full-length + old sequence data set. These rates are in close agreement with the estimated substitution rates found in previous studies from HIV-1 subtypes B and C^4^,^31^.

The usage of older sequences provided rates estimates that were similar to the literature. However, the estimation of the dates of origin and substitution rate from data sets containing old sequence data produced much shorter confidence intervals.

### Dynamic aspects of the southern African epidemic

**Figure 2:**
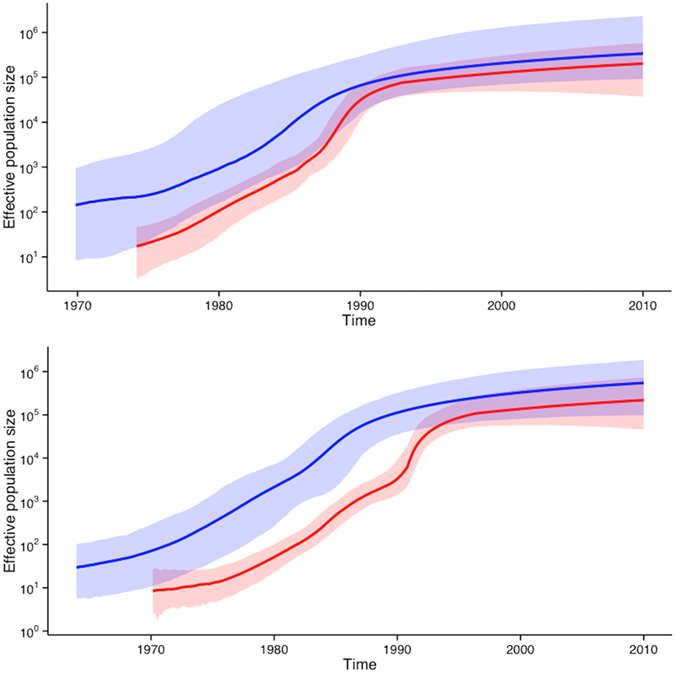
Dynamics growth of the southern African and South African subtype C HIV-1 epidemic as was inferred from Bayesian Skyline Plot reconstruction. On the top are the BSP reconstructions from *gag* p24 sequence data and at the bottom are the BSP reconstructions from *pol* sequence data. Blue graphs represent sequence data from the entire southern African region while red graphs represents data containing only South African isolates. Solid lines depict the mean estimated Ne through time while shaded areas correspond to the 95% highest posterior density intervals.

Epidemic reconstruction was performed from selected parametric runs from all four southern African data sets. A large degree of agreement was observed in the growth of the effective population size (Ne) through time between the *gag* p24 and *pol* data sets and between data sets containing only contemporary strains (full-length data sets) and data sets that were supplemented with old sequence data (Fig. 2).

The *gag* p24 reconstruction suggests a linear increase in the Ne from the start of the HIV-1 epidemic in the southern African region in the early 1960s or late 1950s up till the mid 1970s. This linear increase in the Ne is then followed by an exponential growth phase, which lasted untill the mid 1980s, after which the Ne stabilized. The epidemic reconstruction from the *pol* sequence data sets suggests broadly similar trends (Fig. 2). The Bayesian Skyline Plot reconstruction form data containing only South African sequence information suggests a slow increase in the Ne from the start of the epidemic in the country around 1960 until the start of the 1980s (Fig. 2). This period of slow growth was followed by a rapid increase in the Ne throughout the 1980s and early 1990s and has stabilized in recent years. Epidemic reconstruction performed under SkyRide and SkyGrid models produced similar results (Supplementary Figures S1 and S2).

In addition to the estimation of the Ne through time, the estimated number of lineages through time was also calculated (Table 3). Once again very similar results were observed between methods and different genomic regions. The results indicate a massive increase in the viral diversity within the HIV-1 subtype C epidemic throughout the 1970s and 1980s in the southern African epidemic. In fact, by the start of the 1990s, more than 98% of all currently circulating strains were already present within the region. Similarly, the estimation of the percentage lineages through time (PLTT) from the South African data sets suggests that the largest period of viral expansion occurred during the course of the 1980s with viral variance increasing from ~30.0% of current strains to over 90.0% during that time period (Table 3).

### Relationship between southern African sequences and HIV-1 subtype C pandemic

**Figure 3:**
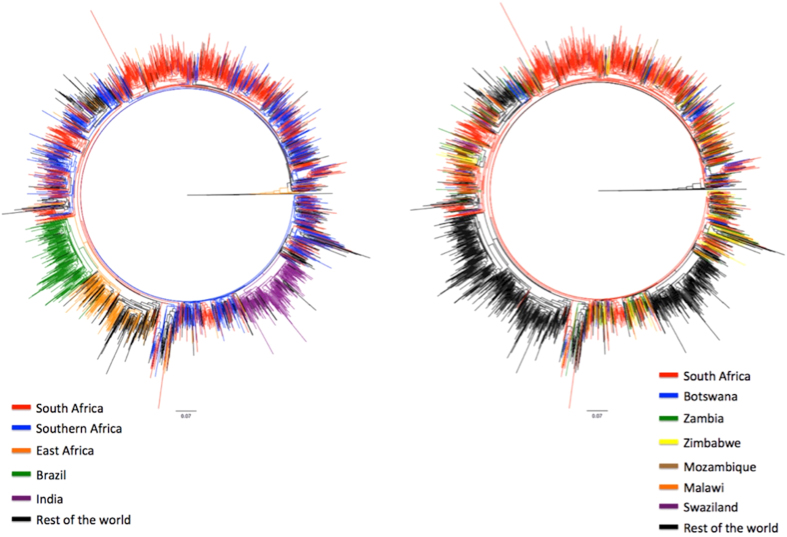
Maximum Likelihood tree topology constructed from *pol* sequence data. The tree contains a total of 2572 taxa and was constructed in phyML, with the use of the HKY85+G (alpha = 0.8) method of nucleotide substitution. The genetic distance is shown in the bottom line and corresponds to the length of the branches. Each of the branches has been colour coded corresponding to the place of origin of each of the taxa. In the first tree southern African countries excluding South Africa has been coloured in blue, while in the second tree individual countries are coloured with their own unique colours.

To place the southern African HIV-1 subtype C epidemic in the broader context of the global HIV-1 subtype C pandemic, large-scale phylogenies were inferred. This was achieved through the construction of large-scale Maximum Likelihood (ML)-tree topologies. HIV-1 subtype C strains from the southern African region presented with a strong panmixia pattern of clustering, indicating multiple introductions of strains in southern African countries (Fig. 3). A strong basal clustering of the old sequences was observed in the tree topologies. We have investigated and described this basal clustering in further detail elsewhere^38^. Detailed analyses of the inferred tree topologies with the PhyloType application confirmed the strong panmixia pattern of subtype C strains from southern African countries. The large ML tree could identify monophyletic clusters containing most of the East-African, Brazilian and Indian sequences. However, for southern Africa there were very few clusters that were supported by bootstrap support >90%. For example, South African HIV-1 subtype C sequences fall in many distinct monophyletic clusters. However, the internal branches of the trees are poorly supported, which makes it difficult to identify distinct viral populations. For example, querying the large tree for viral lineages with support >90 and with more than 20 sequences, we can identify only two lineages from South Africa (the first one with 34 taxa and second with 39 taxa), representing only 9% of the South African sequences within the data set. South Africa, as well as the rest of the southern African region, are experiencing a generalized HIV epidemic with over >10 million infected individuals Even with the usage of >3000 strains, which is still a low sample coverage, we cannot identify specific geographical strains with high confidence. Wertheim and colleagues^39^ also highlighted this problem. Our combined results highlight either that sample coverage needs to increase drastically in order to identify specific strains or that there were a high number of introductions from neighbouring countries, making it difficult to identify country specific strains.

### Discussion and Conclusion

The results presented here places the date of tMRCA of the southern African HIV-1 subtype C epidemic at around 1960 (95% HPD 1956 – 1964), with similar estimates for individual countries within the region. The usage of old archived strains in the present study has greatly enhanced the reconstruction of the epidemic by producing estimates with much smaller confidence intervals (Fig. 1). In the inference presented here it would appear that the estimated dates of origin for the epidemic within the southern African region and the estimates for the epidemic within South Africa are very similar (~1960). This similarity may derive from the fact that the genetic diversity observed amongst South African infected individuals, both from historical sampling as well as from contemporary strains, closely reflects the diversity across the entire southern African region. The large number of genetic variants of subtype C in South Africa can largely be attributed to the extent of migration within the southern African region, with the bulk of migration being directed towards South Africa.

The estimated PLTT from the South African data set suggest a massive introduction of viral strains into the country during the 1980s with viral diversity increasing from <20.0% in 1980 to >90.0% by the 1990s. Given these findings as well as the close panmixia clustering of South African and southern African subtype C strains, it is reasonable to assume that a large number of infections were introduced into South Africa via foreign migrants from other southern African nations during the 1970s and 80s.

A recent study which investigated the spatiotemporal origin of HIV-1 group M in the central African region^40^, demonstrated that the ancestral subtype C strain of HIV-1 migrated from Kinshasa to the southern provinces of the Democratic Republic of the Congo (DRC). This migration occurred via major rail networks connecting Kinshasa with southern DRC. Phylodynamic analyses suggest that the ancestral subtype C strain of HIV-1 was introduced into the southern Katanga region of the DRC in the late 1930s (95% HPD 1919 – 1957) then spread independently to east and southern African respectively, as suggested in the large subtype C phylogenetic tree. This spread was probably facilitated through migrant mine labourers as the bulk of mining activities within the DRC were centred in the mineral rich south of the DRC at the time.

South Africa, as the largest country within the region, saw a massive degree of inward migration during the 20^th^ century. In the mid 1980s, it is estimated that around 1.5 million foreign workers were employed as migrant labourers in the country^41^,^42^, the vast majority of whom were employed in the mining sector. Following the early spread of HIV in southern Africa, in particular the high prevalence of HIV in Malawi during the 1980s, the Apartheid government decided to test all Malawian migrants for HIV from 1987 onwards^43^,^44^. The contribution of migrant labourers from different southern African countries varied substantially through time (Supplementary Table S1). In addition, foreign labourers also included workers from former independent countries within the current confines of the Republic of South Africa (i.e. Transkei, Ciskei, Venda and Bophuthatswana). Migrant workers were considered to be high-risk individuals for infection in the latter part of the 1980s^41^. The role of oscillating migration, (including domestic and foreign sources), and its effect on the HIV epidemic within South African have been well documented to date^45^,^46^,^47^,^48^,^49^,^50^,^51^. This is particularly true for migrant mine workers living in single-sex dormitories for long periods of time. The extreme isolation of workers coupled with prolonged periods of separation from their families, led a large number of migrants seeking multiple sexual partners. The close proximity of commercial sex-workers on the mines further put migrant miners at high risk for infection^52^.

Dynamic reconstruction of the epidemic in the present study shows strong periods of epidemic growth during the 1970s and 1980s for the southern African epidemic. The exponential growth of the Ne observed during the 1970s and 1980s for the southern African region is in accordance with the high prevalence trends observed in the region during the 1980s, with the exception of South Africa, where increase of prevalence started to be noticed in the early 1990s. For example, in 1992 Zimbabwean antenatal prevalence was 12.5%, Zambia 9.4% and South Africa 2%^1^.

Our results indicate that the epidemic entered a period of exponential growth, as observed in the rising Ne, during the 1980s and 1990s in South Africa (Fig. 2). However, it is unclear how accurate the prevalence estimates for South Africa are in the early years prior to the end of Apartheid. This lack of clarity derives from the existence of the homelands system within Apartheid South Africa. The homelands under Apartheid were ruled as independent or quasi-independent states with their own health care systems, actively designed to separate society within geographical units along racial and ethnic lines. It is unclear if prevalence estimates for South Africa during the early years of the epidemic (1981 - 1994) include estimates of people living in these homelands. Demographic statistics for this period are largely derived from national census data, which did not include former independent homelands^53^,^54^. Thus the rising prevalence estimates, derived from antenatal surveys prior to 1994, could possibly have underestimated the prevalence, as they did not cover all of present day South Africa. With the large degree of uncertainty in prevalence trends for South Africa during the 20^th^ century, it is therefore possible that the epidemic could have entered a period of growth anywhere between 1980 and 1990.

In the early years of the HIV epidemic in South Africa, HIV/AIDS cases were largely confined to the large urban centres of the country. It is clear that the epidemic quickly spread to rural areas within the country in the early 1990s. This spread was facilitated by the domestic migrant labour system as well as by the increased mobility of the population within the country following the abolition of the infamous pass law in 1986. The pass law was discriminatory policy of Apartheid South Africa, which required all non-white individuals to carry “passes” within all urban areas. Following the abolition of the law, urbanization accelerated, particularly amongst Native African nationals, due to the lifting of restrictions. The dynamic of urbanization within the South African context is slightly different from other areas of the world as a substantial number of individuals in urban centres (>80.0%) maintain strong links with their rural roots and frequently return to visit family^54^. This strong connection and circular migration further accelerated the early spread of HIV with South Africa from urban to rural communities. This is supported in the mixing of South African strains with the neighbouring countries (Fig. 3).

Some of the potential limitations of our study include the usage of sub-genomic regions in the phylogenetic inference and low sequencing coverage of the epidemic in the region. A better sample coverage and whole genomes may have allowed us to characterize specific introductions of HIV-1 to South Africa. We aim to produce larger data sets in the future as part of a new consortium, the PANGEA_HIV^55^. This data set may identify specific subtype C strains with different epidemic characteristics circulating in the country. Additionally, this rich source of genotypic data, coupled with detailed epidemiological records, can be used to model the HIV epidemic in greater detail through the application of newer coalescent models (i.e. Birth-Death^56^, Fossilized Birth-Death^57^, and Susceptible Infected and Recovered^58^ (SIR) models). PANGEA_HIV is currently investigating the strength and limitations of these methods as part of a global modelling consortium within the context of generalized HIV epidemics in sub-Saharan Africa.

In conclusion, our results shed light on the epidemic origin and history of the southern African and South African HIV-1 subtype C epidemics. The origin of the subtype C epidemic in the region can be placed around 1960 (95% HPD 1956 – 1964) with strong periods of epidemic growth during the 1970s and 80s for the southern African region and during the 1980s and 90s for South Africa. The periods of strong epidemic growth coincide with periods of socio-political changes in the region during the latter part of the 20^th^ century. The results from the phylogenetic reconstruction support that migration played an important role in facilitating the introduction and spread of the virus throughout the region and in South Africa in particular. Furthermore, the usage of older sequences provided more accurate estimates of the origin and rate of growth of the epidemic than previous subtype C studies, which used mostly contemporary sequences, further reducing uncertainty in our estimates.

## Additional Information

**How to cite this article**: Wilkinson, E. *et al.* History and origin of the HIV-1 subtype C epidemic in South Africa and the greater southern African region. *Sci. Rep.* **5**, 16897; doi: 10.1038/srep16897 (2015).

## Supplementary Material

Supplementary Information

## Figures and Tables

**Table 1:** Composition of longitudinal data sets, which were used in the epidemic reconstruction of the South African and southern African epidemics.

Data set	Country	1989~1993	1994~1999	2000~2007	2008~present
*gag*.full-length	Botswana		23	27	1
	Zambia	1	1	11	2
	Malawi	1		7	6
	South Africa	1	16	273	6
*gag*.full-length + old	Botswana		23	27	1
	Zambia	3	11	11	2
	Malawi	1		7	6
	South Africa	1	36	273	6
	South Africa*	26	36	273	6
*pol*.full-length	Botswana		23	27	1
	Zambia	1	1	11	2
	Malawi	1		7	6
	South Africa	1	36	273	6
*pol*.full-length + old	Botswana		23	27	1
	Zambia	1	5	11	2
	Malawi	1		7	6
	South Africa	1	36	273	6
	South Africa*	46	36	273	6

The asterisk (*) indicates data sets inclusive of newly genotyped samples.

**Table 2:** Estimated dates of origin for the four different southern African data sets under various models and parameters.

Models and parameters	*gag* p24 full-length strains					*gag* p24 full-length strains + old archived strains				
	Mean	Median	95% HPD lower	95% HPD upper	ESS	Mean	Median	95% HPD lower	95% HPD upper	ESS
Relax.bsp.est	1951,8	1951,7	1963,1	1940,7	1990,9	1961,2	1961,7	1966,3	1955,1	452,0
Relax.bsp.fix	1963,3	1963,6	1968,6	1957,6	493,4	1963,1	1963,6	1968,2	1957,0	292,1
Relax.const.est	1967,8	1968,6	1974,9	1958,8	1116,2	1962,2	1962,6	1967,3	1956,1	372,1
Relax.const.fix	**1960,4**	**1961,3**	**1968,5**	**1950,9**	**758,9**	1959,0	1960,1	1967,2	1948,1	358,8
Relax.expo.est	1948,4	1948,2	1956,2	1941,0	202,5	**1959,0**	**1959,8**	**1963,5**	**1954,6**	**368,7**
Relax.expo.fix	1962,5	1962,7	1966,5	1958,5	229,8	1961,9	1962,0	1965,6	1958,0	340,8
Strict.bsp.est	1949,2	1949,1	1960,5	1938,7	1571,4	1959,9	1960,5	1968,2	1950,3	1275,6
Strict.bsp.fix	1963,0	1963,3	1968,0	1956,9	794,6	1962,8	1963,1	1967,7	1957,1	1595,0
Strict.const.est	1961,6	1962,3	1973,9	1948,6	303,1	1960,7	1961,3	1969,0	1951,1	330,4
Strict.const.fix	1962,0	1963,5	1969,6	1950,5	319,9	1961,7	1962,2	1968,3	1954,1	349,1
Strict.expo.est	1957,2	1958,9	1968,3	1943,8	336,8	1961,6	1961,9	1966,9	1955,8	332,8
Strict.expo.fix	1962,6	1962,7	1966,4	1958,8	336,8	1961,5	1961,6	1965,5	1957,6	226,0
	***pol* full-length strains**					***pol* full-length strains + old archived strains**				
Relax.bsp.est	1957,9	1958,0	1966,9	1948,4	2720,1	**1958,3**	**1959,1**	**1961,4**	**1955,3**	**1342,6**
Relax.bsp.fix	1958,3	1958,5	1964,2	1951,3	3791,7	1954,7	1955,0	1963,5	1946,1	429,6
Relax.const.est	1956,7	1956,8	1964,3	1948,6	2978,4	1981,4	1984,1	1988,4	1967,5	214,8
Relax.const.fix	**1958,4**	**1959,2**	**1964,9**	**1951,8**	**1784,9**	1986,5	1987,1	1988,7	1982,1	257,8
Relax.expo.est	1957,5	1957,6	1964,5	1949,9	2818,8	1958,3	1958,7	1965,2	1951,4	1361,4
Relax.expo.fix	1964,7	1964,8	1967,6	1961,9	779,7	1957,9	1958,3	1964,6	1950,1	1596,4
Strict.bsp.est	1958,8	1959,0	1964,5	1953,1	750,0	1952,1	1952,4	1959,6	1944,3	1618,5
Strict.bsp.fix	1958,7	1958,9	1964,4	1952,7	781,2	1952,3	1952,6	1959,3	1944,1	1677,7
Strict.const.est	1958,2	1958,5	1964,6	1951,0	1275,2	1959,7	1959,7	1964,5	1954,9	295,1
Strict.const.fix	1954,2	1954,4	1959,9	1948,2	700,7	1959,1	1959,1	1964,3	1954,7	202,5
Strict.expo.est	1957,2	1957,0	1962,6	1952,4	495,2	1959,6	1959,5	1964,1	1954,9	266,7
Strict.expo.fix	1963,9	1963,9	1966,8	1961,0	461,3	1955,5	1955,7	1963,9	1946,9	1206,5

The mean, median, the 95% HPD, and the ESS of each run are shown. The “best fitting runs” as determined by BF comparison are highlighted in bold.

**Table 3:** Estimated percentage lineages through time for the four different southern African data sets.

Time	Southern Africa Percentage Linages Through Time							
	*gag* p24 full-length + old sequence				*pol* full-length + old sequence			
	Mean	Median	Upper	Lower	Mean	Median	Upper	Lower
2010	100	100	100	100	100	100	100	100
2005	100	100	100	100	100	100	100	100
2000	99.76	99.73	100	99.47	99.66	99.73	100	99.47
1995	99.27	99.2	99.47	98.67	99.42	99.47	99.47	99.2
1990	98.57	98.67	99.47	97.6	98.43	98.4	99.2	97.33
1985	95.89	96.27	98.4	91.2	91.77	91.98	95.99	86.1
1980	61.29	61.07	77.87	46.93	54.3	54.28	66.58	41.98
1975	23.57	22.93	37.33	12.53	9.39	9.09	16.31	4.28
1970	8.46	7.47	18.67	2.93	1.62	1.6	2.67	0.8
1965	2.47	2.4	5.33	0.8	0.75	0.8	1.07	0.53
	**South Africa Percentage Linages Through Time**							
2010	100	100	100	100	100	100	100	100
2005	100	100	100	100	100	100	100	100
2000	97.35	99.89	100	98.32	99.84	100	100	99.41
1995	96.75	95.09	98.61	97.16	99.23	99.41	99.71	98.24
1990	94.52	93.13	98.32	90.78	96.94	97.36	99.41	91.79
1985	70.23	70.69	90.49	42.34	72.03	73.9	91.5	42.82
1980	31.2	27.77	70.19	8.12	32	29.03	70.97	8.21
1975	8.98	6.45	32.19	1.45	9.21	6.74	32.55	1.47
1970	2.2	1.4	8.41	0.58	2.26	1.47	8.5	0.59
1965	0.55	0.35	2.1	0.15	0.56	0.37	2.13	0.15

These estimates were derived from non-parametric runs under a relaxed molecular clock assumption and an estimated substitution rate.
