# Supplementary material for: History and origin of the HIV-1 subtype C epidemic in South Africa and the greater southern African region
Source: Sci Rep. 2015 Nov 17;5:16897. doi: 10.1038/srep16897 (PMC4648088; doi:10.1038/srep16897)

Supplementary information

History and origin of the HIV-1 subtype C epidemic in South Africa and the greater southern African region

E Wilkinson, S Engelbrecht, T de Oliveira

Table of content

	Page
Supplementary Table 1	2
Supplementary Figure 1	3
Supplementary Figure 2	4

Supplementary Table 1: Contribution of foreign migrant labourers per country working in South Africa gold mines between 1920 and 1990.

Year	Angola	Botswana	Lesotho	Malawi	Mozambique	Swaziland	Tanzania	Zambia	Zimbabwe	Other	Total
1920	0	2112	10439	354	77921	3449	0	12	179	5484	99950
1925	0	2547	14256	136	73210	3999	0	4	68	14	94234
1930	0	3151	22306	0	77828	4345	183	0	44	5	107862
1935	0	7505	34788	49	62576	6865	109	570	27	9	112498
1940	698	14427	52044	8037	74693	7152	0	2725	8112	70	167958
1945	8711	10102	36414	4973	78588	5688	1461	27	8301	4732	158997
1950	9767	12390	34467	7831	86246	6619	5495	3102	2073	4826	172816
1955	8801	14195	36332	12407	99449	6682	8758	3849	162	2299	192934
1960	12364	21404	48842	21934	101733	6623	14025	5292	747	844	233808
1965	11169	23630	54819	38580	89191	5580	404	5898	653	2686	232610
1970	4125	20461	63988	78492	93203	6269	0	0	3	972	267513
1975	3431	20291	78114	27904	97216	8391	0	0	2485	12	237844
1980	5	17763	96309	13569	39539	8090	0	0	5770	1404	182449
1985	0	18079	97639	16849	50126	12365	0	0	0	4	195062
1990	0	15720	108780	72	50104	17816	0	0	2	0	192494

Supplementary Figure 1: Dynamics growth of the southern African and South African HIV-1 subtype C epidemics inferred from selected Bayesian SkyRide runs. On the top are the BSP reconstructions from *gag* p24 sequence data and at the bottom are the BSP reconstructions from *pol* sequence data. Blue graphs represent sequence data from the entire southern African region while red graphs represents data containing only South African isolates. Solid lines depict the mean estimated Ne through time while shaded areas correspond to the 95% highest posterior density intervals.

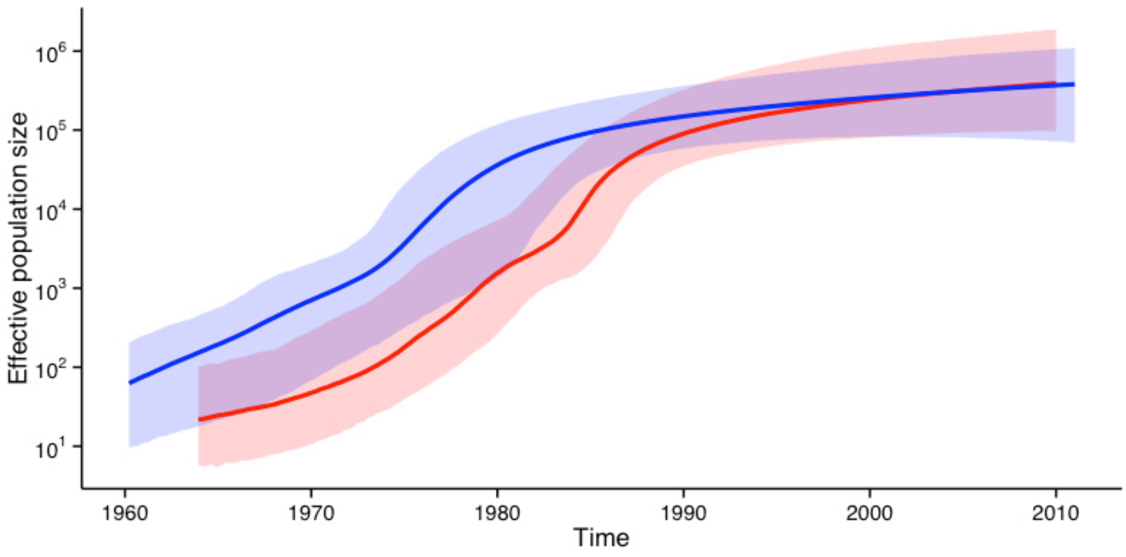

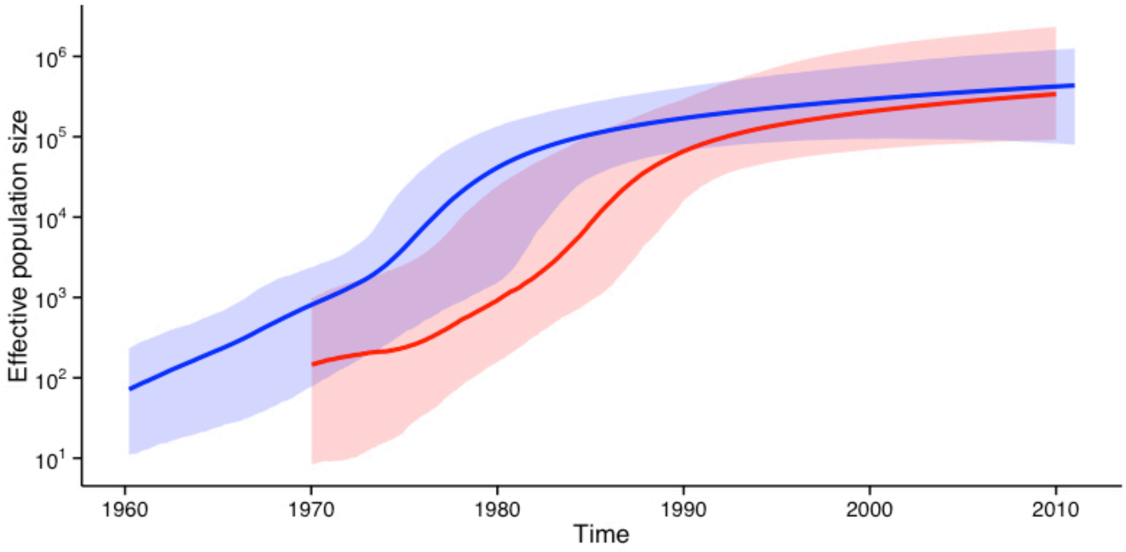

Supplementary Figure 2: Dynamics growth of the southern African and South African HIV-1 subtype C epidemics inferred from selected Bayesian SkyGrid runs. On the top are the BSP reconstructions from *gag* p24 sequence data and at the bottom are the BSP reconstructions from *pol* sequence data. Blue graphs represent sequence data from the entire southern African region while red graphs represents data containing only South African isolates. Solid lines depict the mean estimated Ne through time while shaded areas correspond to the 95% highest posterior density intervals.

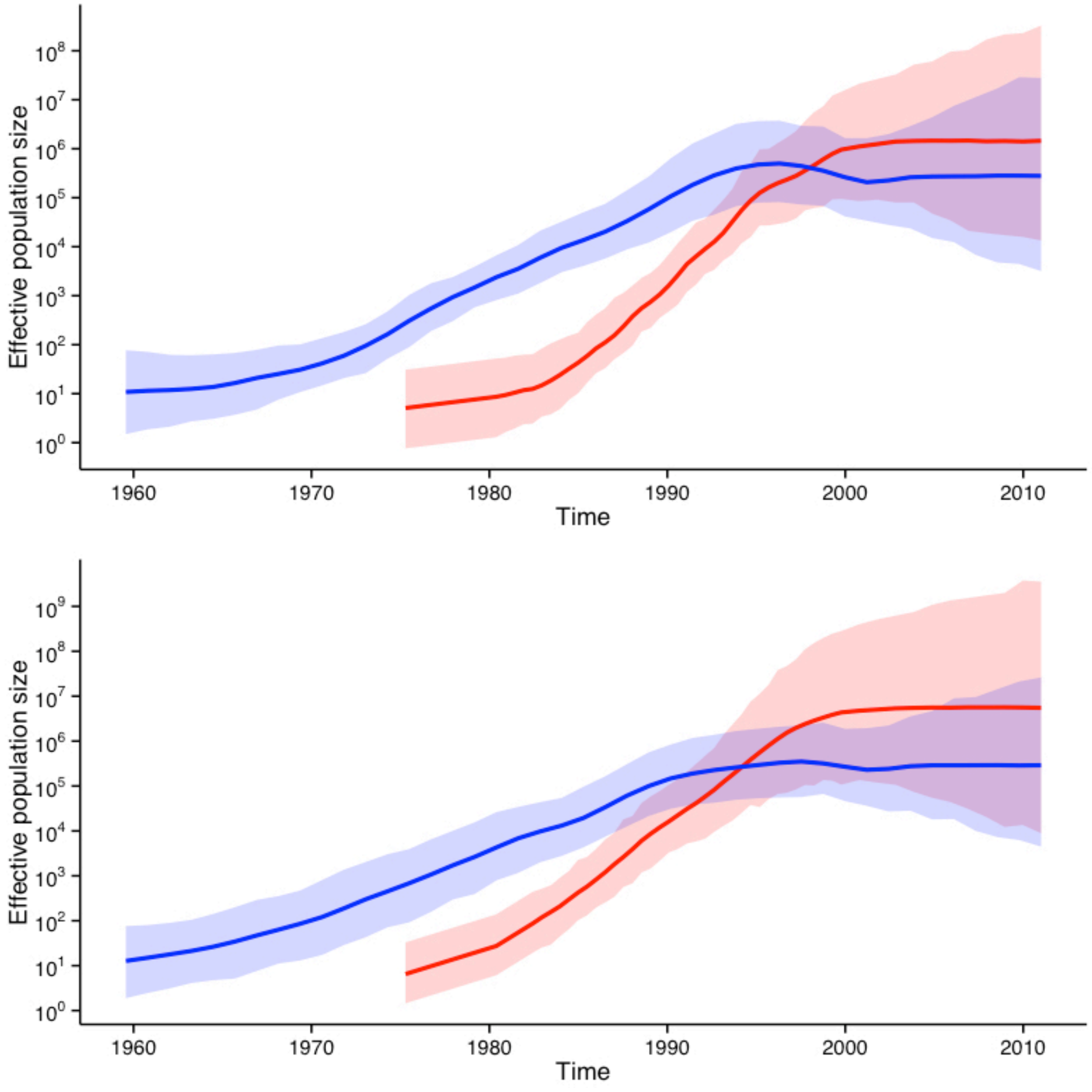

Supplement: Supplementary Information [file srep16897-s1.pdf]
